# A CpG 1018 adjuvanted neuraminidase vaccine provides robust protection from influenza virus challenge in mice

**DOI:** 10.1038/s41541-022-00486-w

**Published:** 2022-07-22

**Authors:** Shirin Strohmeier, Fatima Amanat, John D. Campbell, Paula Traquina, Robert L. Coffman, Florian Krammer

**Affiliations:** 1 Department of Microbiology, Icahn School of Medicine at Mount Sinai, New York, NY USA; 2 Department of Biotechnology, University of Natural Resources and Life Sciences, Vienna, Austria; 3 Graduate School of Biomedical Sciences, Icahn School of Medicine at Mount Sinai, New York, NY USA; 4 Dynavax Technologies, Emeryville, CA USA; 5 Department of Pathology, Molecular and Cell-Based Medicine, Icahn School of Medicine at Mount Sinai, New York, NY USA

**Keywords:** Protein vaccines, Protein vaccines

## Abstract

Influenza virus infections pose a significant threat to global health. Vaccination is the main countermeasure against influenza virus spread, however, the effectiveness of vaccines is variable. Current seasonal influenza virus vaccines mostly rely on the immunodominant hemagglutinin (HA) glycoprotein on the viral surface, which usually leads to a narrow and strain-specific immune response. The HA undergoes constant antigenic drift, which can lead to a dramatic loss in vaccine effectiveness, requiring the annual reformulation and readministration of influenza virus vaccines. Recently, it has been demonstrated that the subdominant glycoprotein, neuraminidase (NA), is an attractive target for vaccine development. Here, we tested a newly developed recombinant influenza virus N1 neuraminidase vaccine candidate, named N1-MPP, adjuvanted with CpG 1018, a TLR9 agonist. Additionally, N2-MPP and B-NA-MPP vaccine constructs have been generated to cover the range of influenza viruses that are seasonally circulating in humans. These constructs have been characterized in vitro and in vivo regarding their functionality and protective potential. Furthermore, a trivalent NA-MPP mix was tested. No antigenic competition between the individual NA constructs was detected. By adjuvating the recombinant protein constructs with CpG 1018 it was possible to induce a strong and robust immune response against the NA, which provided full protection against morbidity and mortality after high lethal challenges in vivo. This study provides important insights for the development of a broadly protective NA-based influenza virus vaccine candidate.

## Introduction

Influenza viruses can cause severe respiratory infections in humans and pose a significant threat to global health. According to the World Health Organization, seasonal influenza viruses cause up to five million cases of severe influenza virus infection, including up to 650,000 deaths each year globally. The influenza virus contains two glycoproteins on its viral surface, which include the immunodominant hemagglutinin (HA) and the immunosubdominant neuraminidase (NA). Current available seasonal influenza virus vaccines mostly target the HA as the NA content in the vaccines is not standardized and can vary significantly. While the HA of the influenza virus is known to induce a strong neutralizing antibody response in humans, it is more susceptible to antigenic drift. This can lead to a mismatch between vaccine strains and circulating strains, resulting in a significant loss of effectiveness of the annual vaccines.

Over the past few years, the NA has emerged as an attractive target for vaccine development since it is less prone to undergo antigenic drift and therefore is antigenically more stable. In addition, it has been demonstrated in animal models as well as in humans that anti-NA immunity correlates with protection and reduces viral shedding^1–5^. The main obstacles to using NA as a vaccine antigen are its lack of standardization in seasonal vaccine preparations as well as its fragile stability. Indeed, the amount of NA in seasonal vaccines usually varies in quality and quantity^6^ and it is likely that the structural integrity in current vaccine formulations is suboptimal. Additionally, it has been hypothesized that antigenic competition occurs between HA and NA in vaccine formulations, making the NA immunosubdominant^7^. The anti-NA immunity acquired after vaccination with live attenuated or inactivated vaccines is mediocre at best^8^. Stable, recombinant NA protein has been shown to be immunogenic and protective in animal models^5,6,9^ and may enhance NA-based immunity in humans by standalone administration or as an admixture to seasonal influenza virus vaccines. Recently, we have developed a recombinant NA vaccine candidate, named N1-MPP^10^. This vaccine candidate utilized the tetramerization domain of the phosphoprotein of the measles virus to multimerize and stabilize the NA protein. N1-MPP can form fully enzymatically active NA tetramers which are highly protective in vivo in a mouse model and induce high titers of neuraminidase inhibiting (NI) antibodies after vaccination. The tetramerization of the protein is a crucial factor in generating an NA-based vaccine since it has been previously shown that only multimeric but not monomeric protein confers protection in vivo^11,12^.

In general, the efficiency of a vaccine depends on the magnitude, duration, and quality of the immune response that is induced. However, recombinant protein vaccines usually tend to induce a lower immune response compared to whole virus vaccines due to the lack of molecules that engage innate immune receptors. Here, we tested N1-MPP—as well as novel N2-MPP and B-NA-MPP constructs in a trivalent formulation—adjuvanted with the GMP-produced TLR9 agonist CpG 1018 (ODN1018) in the mouse model. CpG 1018 is currently used in the licensed hepatitis B virus vaccine HEPLISAV-B®^13,14^. The work described here was performed in preparation for the clinical testing of N1-MPP with CpG 1018.

## Results

### A prime-boost regimen with N1-MPP is required to achieve full protection in a naive mouse model

**Fig. 1:**
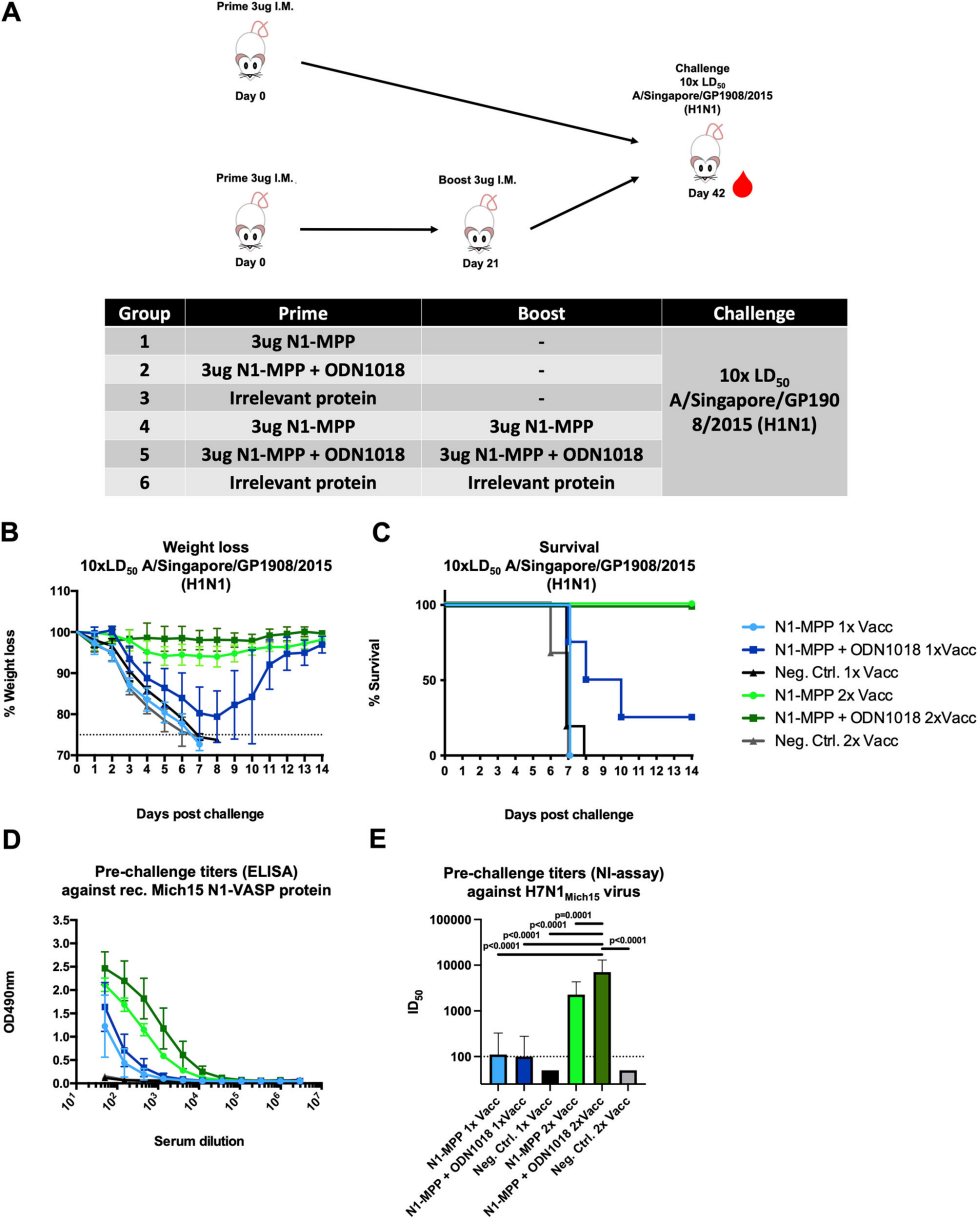
Proof of principle experiment to assess if one vaccination is sufficient to induce a robust and protective immune response in vivo. **A** Mice (*n* = 5 per group, except for Neg. Contr. 2× Vacc where *n* = 3) were vaccinated either once (prime) or twice (prime-boost) with 3 μg of N1-MPP, 3 μg N1-MPP + ODN1018 or irrelevant protein following a challenge with 10xmLD_50_ of A/Singapore/GP1908/15 H1N1 (IVR-180). Weight loss and survival were monitored over a 14-day time period. Mice were bled on day 21 and 42 post prime for serological analysis. **B** Weight loss curve (mean plus standard deviation) and **C** survival after challenge with 10xmLD_50_ of A/Singapore/GP1908/15 H1N1 (IVR-180) are shown. Differences in survival between the vaccine groups and respective control groups as well as between matched prime-only and prime-boost groups were analyzed using a Mantel–Cox log-rank test. N1-MPP + ODN1018 1× Vacc vs. Neg. Contr. 1× Vacc *p* = 0.0298; N1-MPP 1× Vacc vs. N1-MPP + ODN1018 1× Vacc *p* = 0.0143; N1-MPP 2× Vacc vs Neg. Contr. 2× Vacc *p* = 0.0067; N1-MPP + ODN1018 2× Vacc vs. Neg. Contr. 2× Vacc *p* = 0.0067; N1-MPP 1× Vacc vs. N1-MPP 2× Vacc *p* = 0.0027; N1-MPP + ODN1018 1× Vacc vs. N1-MPP + ODN1018 2× Vacc *p* = 0.0494. The difference between the remaining curves were not statistically significant *p* > 0.05 or they were not compared. **D** ELISA titers in serum pre-challenge are shown (mean plus standard deviation). **E** NI titers 42 days post prime using H7N1_Mich15_ virus (geometric mean plus standard deviation of the geometric mean). Statistical analysis in **E** was performed using a one-way ANOVA corrected for multiple comparisons.

To assess the adjuvant effect of CpG 1018 on the N1-MPP antigen in a prime-only or prime-boost vaccination regimen, naive female 6–8 week old BALB/c mice (*n* = 5 per group) were either vaccinated once with 3 μg N1-MPP, 3 μg N1-MPP + 3 μg CpG 1018, or 3 μg of an irrelevant protein, or twice with the same formulations in a 3-week interval (Fig. 1A). Three weeks post boost, mice were then challenged with 10 x the 50% mouse lethal dose (mLD_50_) of A/Singapore/GP1908/15 H1N1 (IVR-180, this virus is antigenically equivalent to A/Michigan/45/15) virus, and weight loss and survival were monitored over a 14-day period. As shown in Fig. 1B, mice that only received one vaccination with non-adjuvanted N1-MPP or irrelevant protein all succumbed to infection around day 8 post challenge. Mice vaccinated once with N1-MPP + CpG 1018 experienced high weight loss (approximately 20%) and 4 out of 5 mice succumbed to infection around day 10 post challenge. Nevertheless, mice vaccinated with the adjuvanted formulation survived significantly longer (*p* = 0.0143). However, groups of mice that received two vaccinations of N1-MPP or N1-MPP + CpG 1018 did not experience any weight loss and all animals in these groups survived the challenge. All mice in the negative control group succumbed to infection around day 8 (Fig. 1B, C). To assess serological characteristics of the serum antibodies induced after vaccination, the serum was tested via an enzyme-linked immunosorbent assay (ELISA) against recombinant Mich15 N1-VASP protein. This protein containing a different tetramerization domain was used to avoid the detection of antibodies induced against the MPP tetramerization domain. After the prime, only a low antibody response against the NA was detectable, with slightly higher levels in the N1-MPP + CpG 1018 group (Fig. 1D). Serum from mice vaccinated with the prime-boost regimen showed a strong increase in N1-specific antibodies, with N1-MPP + CpG 1018 performing the best (Fig. 1D). The same trend was observed in a NI assay using an H7N1_Mich15_ virus, which contains an irrelevant HA and the N1 of A/Michigan/45/15. Mice vaccinated with the prime-boost regimen showed high levels of NI active antibodies in their serum with the N1-MPP + CpG 1018 group performing best (geometric mean 50% inhibitory dilution (ID_50_) = 7046) indicating that the CpG 1018 adjuvant leads to a more robust immune response to the antigen (Fig. 1E). The NI results also indicated a statistically significant difference between adjuvanted and non-adjuvanted groups in the prime-boost regimen.

### Passive serum transfer from vaccinated to naive mice protects from lethal challenge

**Fig. 2:**
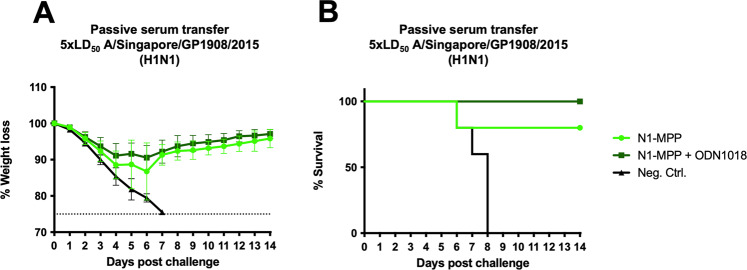
Passive transfer of N1-MPP sera. BALB/c mice (*n* = 5 per group) received a passive serum transfer intraperitoneally and were challenged with 5xmLD_50_ of A/Singapore/GP1908/15 H1N1 (IVR-180). Weight loss **A** was monitored over a 14-day period (shown is mean plus standard deviation) and the survival **B** of each group is also shown. Differences in survival were analyzed using a Mantel–Cox log-rank test. N1-MPP vs Neg. Ctrl. *p* = 0.0419, N1-MPP + ODN1018 vs Neg. Ctrl. *p* = 0.0034, the remaining differences were not statistically significant (*p* > 0.05).

Serum obtained from mice vaccinated with the prime-boost regimen with N1-MPP, N1-MPP + 3 μg CpG 1018 or an irrelevant protein was used to perform a passive serum transfer into 6–8 week old naive BALB/c mice (*n* = 5 per group) followed by a challenge with 5xmLD_50_ of A/Singapore/GP1908/15 H1N1 (IVR-180). Weight loss and survival were monitored over a 14-day period. Mice which received N1-MPP serum showed ~10% weight loss, with one mouse succumbing to infection on day 6 post challenge. Mice that received N1-MPP + CpG 1018 experienced slightly less weight loss than the N1-MPP group and experienced no mortality (Fig. 2A, B). The negative control group succumbed to infection by day 8 post challenge.

### CpG 1018 adjuvantation partially breaks the immunodominance of HA over NA in a QIV + rNA formulation in terms of anti-NA antibody titers

**Fig. 3:**
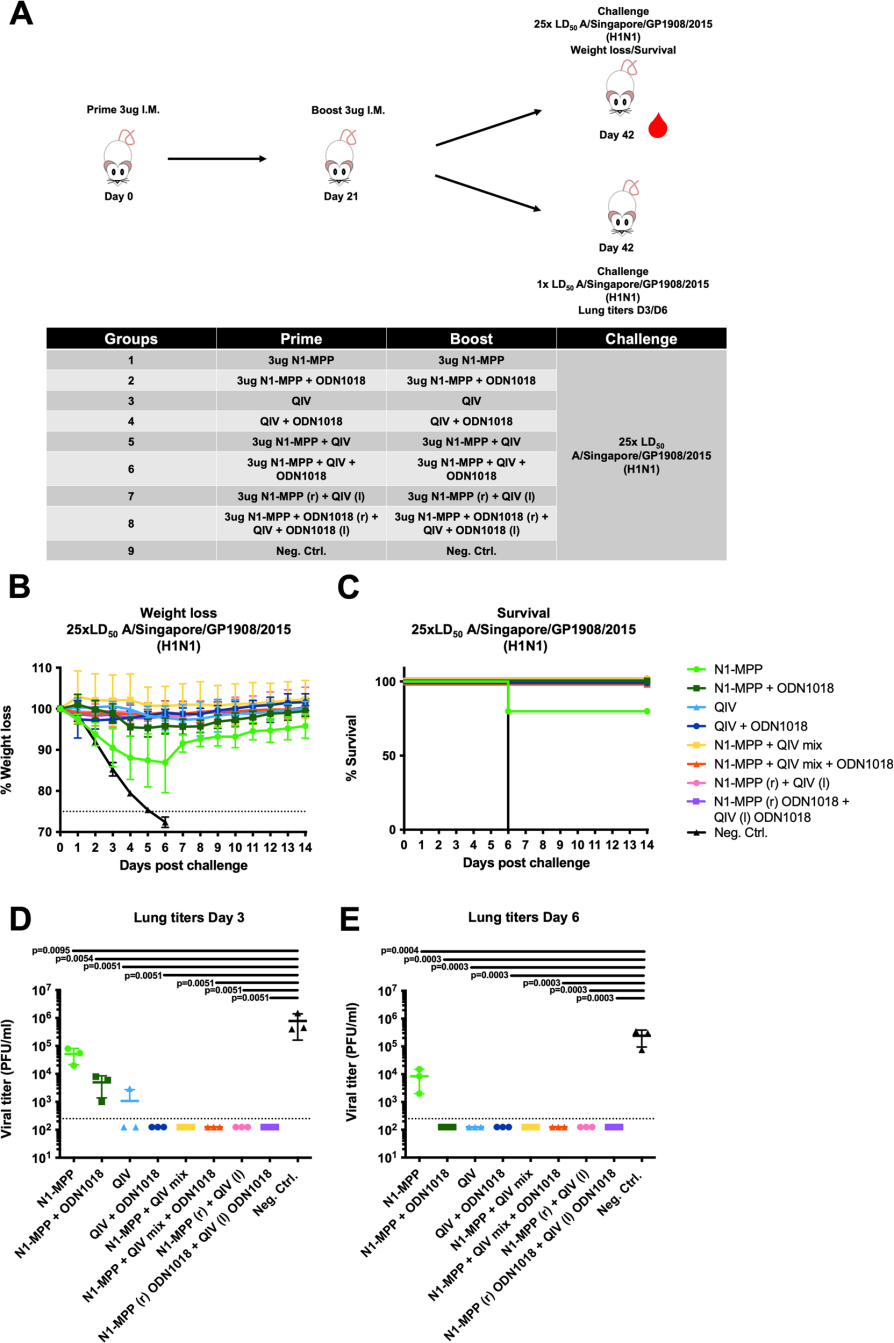
Preclinical assessment of N1-MPP and seasonal QIV in combination with CpG 1018. **A** Vaccination scheme, mice (*n* = 5 per group) were vaccinated in a prime/boost regimen and then challenged either with 25xmLD_50_ of A/Singapore/GP1908/15 H1N1 (IVR-180) to monitor weight loss and survival over a 14-day period or with 1xmLD_50_ of A/Singapore/GP1908/15 H1N1 (IVR-180) to determine viral lung titers. **B** Weight loss curve (mean plus standard deviation) and **C** survival after viral challenge are shown. Differences in survival were analyzed using a Mantel–Cox log-rank test. N1-MPP vs Neg. Ctrl. *p* = 0.0404, all other groups vs. Neg. Ctrl. *p* = 0.0082, all other comparisons were not statistically significant (*p* > 0.05). **D** Viral lung titers obtained on day 3 (*n* = 3 per group) and **E** day 6 (*n* = 3 per group) post challenge. Statistical analysis in **D** and **E** was performed using an one-way ANOVA corrected for multiple comparisons, shown is mean plus standard deviation.

We have previously shown, that – while recombinant NA on its own is immunogenic—admixture to QIV leads to reduced immunogenicity, likely due to the immunodominance of HA over NA^10^. To test if CpG 1018 would improve NA immunogenicity, even in a mixture with QIV, naive female 6–8 week old BALB/c mice (*n* = 5 per group) were vaccinated IM in a prime-boost regimen. Mice received either 3 µg of N1-MPP, 3 µg N1-MPP + 3 μg CpG 1018, QIV (matched with the challenge virus), QIV + 3 μg CpG 1018, 3 µg N1-MPP admixed with QIV, 3 µg N1-MPP admixed with QIV and 3 μg CpG 1018, 3 µg of N1-MPP given in the right leg and QIV at the same time in the left leg (N1-MPP (r) + QIV (l)), 3 µg of N1-MPP + 3 μg CpG 1018 given in the right leg and QIV + 3 μg CpG 1018 given in the left leg at the same time (N1-MPP + 3 μg CpG 1018 (r) + QIV + 3 μg CpG 1018 (l)) or 3 µg of irrelevant protein (Fig. 3A). Following vaccination, mice were challenged with 25xmLD_50_ of A/Singapore/GP1908/2015 H1N1 (IVR-180). Weight loss and survival was monitored over a 14-day period. The non-adjuvanted N1-MPP group showed the highest post challenge weight loss of ~10% and one mouse in the group succumbed to infection on day 6 post infection. All mice in the negative control group succumbed to infection on day 6 post infection. The remaining groups did not experience any morbidity or mortality (Fig. 3B, C).

To observe the effect of CpG 1018 on the reduction of viral load in the lungs, a subset of mice was infected with a lower challenge dose of 1xmLD_50_ A/Singapore/GP1908/15 H1N1 (IVR-180). Lungs were extracted on day 3 (Fig. 3D) and day 6 (Fig. 3E) post infection and the viral titers were determined. On day 3, the negative control group showed the highest viral titer (7.8 × 10^5^ pfu/ml), followed by the N1-MPP group (5.1 × 10^4^ pfu/ml), the N1-MPP + CpG 1018 (5.0 × 10^3^ pfu/ml) and the QIV groups (1.0 × 10^3^ pfu/ml). In the remaining groups, no virus was detectable. On day 6, the virus cleared out in the N1-MPP + CpG 1018 group as well as in the QIV group. The viral titer remained detectible in the N1-MPP group (8.5 × 10^3^ pfu/ml) but was lower than in the negative control group (2.4 × 10^5^ pfu/ml).

**Fig. 4:**
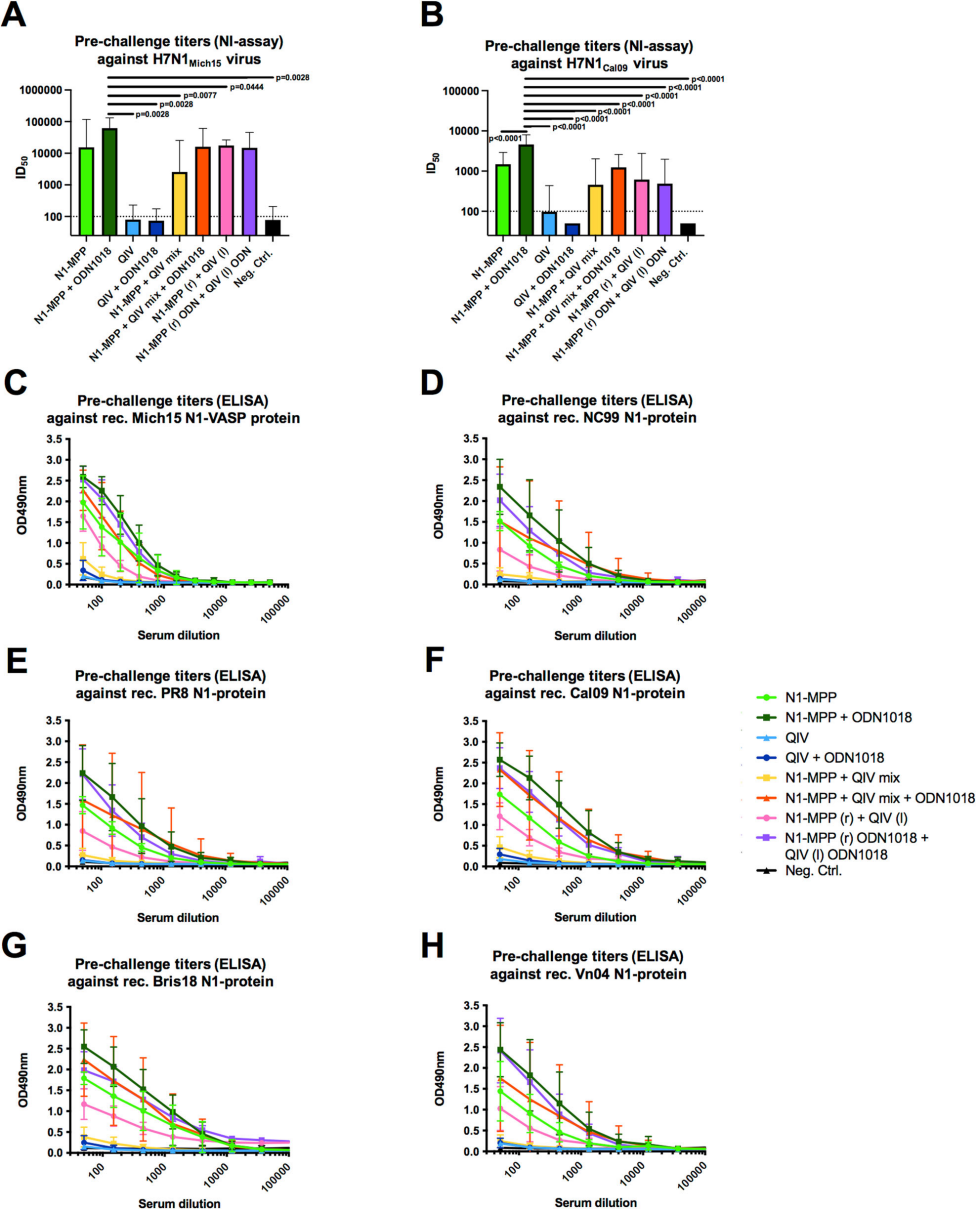
Crossreactivity of serum antibodies against different N1 proteins in NI and ELISA. **A** NI using a reassortant H7N1_Mich15_ virus containing the N1 of A/Michigan/45/2015 or **B** containing the N1 of A/California/04/09. Geometric mean plus standard deviation is shown. Statistical analysis in **A** and **B** was performed using an one-way ANOVA corrected for multiple comparisons, shown is mean plus standard deviation. ELISA crossreactivity testing against **C** Mich15 N1-VASP protein, **D** NC99 N1-VASP protein, **E** PR8 N1-VASP protein, **F** Cal09 N1-VASP protein, **G** Bris18 N1-VASP protein, and **H** Vn04 N1-VASP protein. For **C** to **H**, mean and standard deviation are shown. *N* = 5 per group.

Since it is known that high titers of NI active antibodies correlate with reduction of viral replication as well as a less severe disease outcome, we wanted to assess the level of NI active antibodies induced after vaccination with N1-MPP and QIV in combination with CpG 1018. NI assays were performed with H7N1_Mich15_ virus, which contains the matching NA component to the vaccine antigen, and with H7N1_Cal09_ virus to observe if cross-reactive NI antibodies can be induced. Against H7N1_Mich15_, N1-MPP + CpG 1018 induced the highest level of NI antibodies (ID_50_ = 62309), followed by N1-MPP + CpG 1018 (r) and QIV + CpG 1018 (l) (ID_50_ = 17602), N1-MPP + QIV + CpG 1018 admixed (ID_50_ = 15954) and unadjuvanted N1-MPP (ID_50_ = 15409) (Fig. 4A). Of note, the N1-MPP + CpG 1018, N1-MPP + CpG 1018 (r) and QIV + CpG 1018 (l) and N1-MPP + QIV + CpG 1018 group NI activity were not significantly different suggesting that both administration of HA and NA containing vaccine in different limbs as well as co-administration in the presence of adjuvant may induce similar immune responses while this is not the case when recombinant NA is admixed with QIV without adjuvant. In case of the heterologous H7N1_Cal09_ virus, N1-MPP + CpG 1018 had the highest NI inhibition potential (ID_50_ = 4585), followed by the unadjuvanted N1-MPP group (ID_50_ = 1479) (Fig. 4B).

We also wanted to test if antibodies induced through vaccination with N1-MPP would induce N1 subtype cross-reactive antibodies. The serum was tested in ELISAs against recombinant Mich15 N1-VASP protein (Fig. 4C) matching the vaccine antigen strain, and against NC99 N1 (pre-pandemic N1, Fig. 4D), PR8 N1 (prototype N1, Fig. 4E), Cal09 N1 (Fig. 4F), Bris18 N1 (Fig. 4G) and Vn04 N1 (avian N1, Fig. 4H). Overall, the same trend was seen as in the NI assay, with N1-MPP + CpG 1018 performing the best, followed by N1-MPP + ODN1018 (r) and QIV + CpG 1018 (l) and N1-MPP + QIV + CpG 1018 admixed. This underscores that CpG 1018 is beneficial in inducing a strong and robust antibody response, which appears to be cross-reactive within the N1 subgroup. N1-MPP + QIV admixed induced only a low N1-specific antibody response as well as lower titers of NI active antibodies, underlining the antigenic competition between HA and NA.

### Antigen dose de-escalation shows a clear adjuvant effect of CpG 1018

**Fig. 5:**
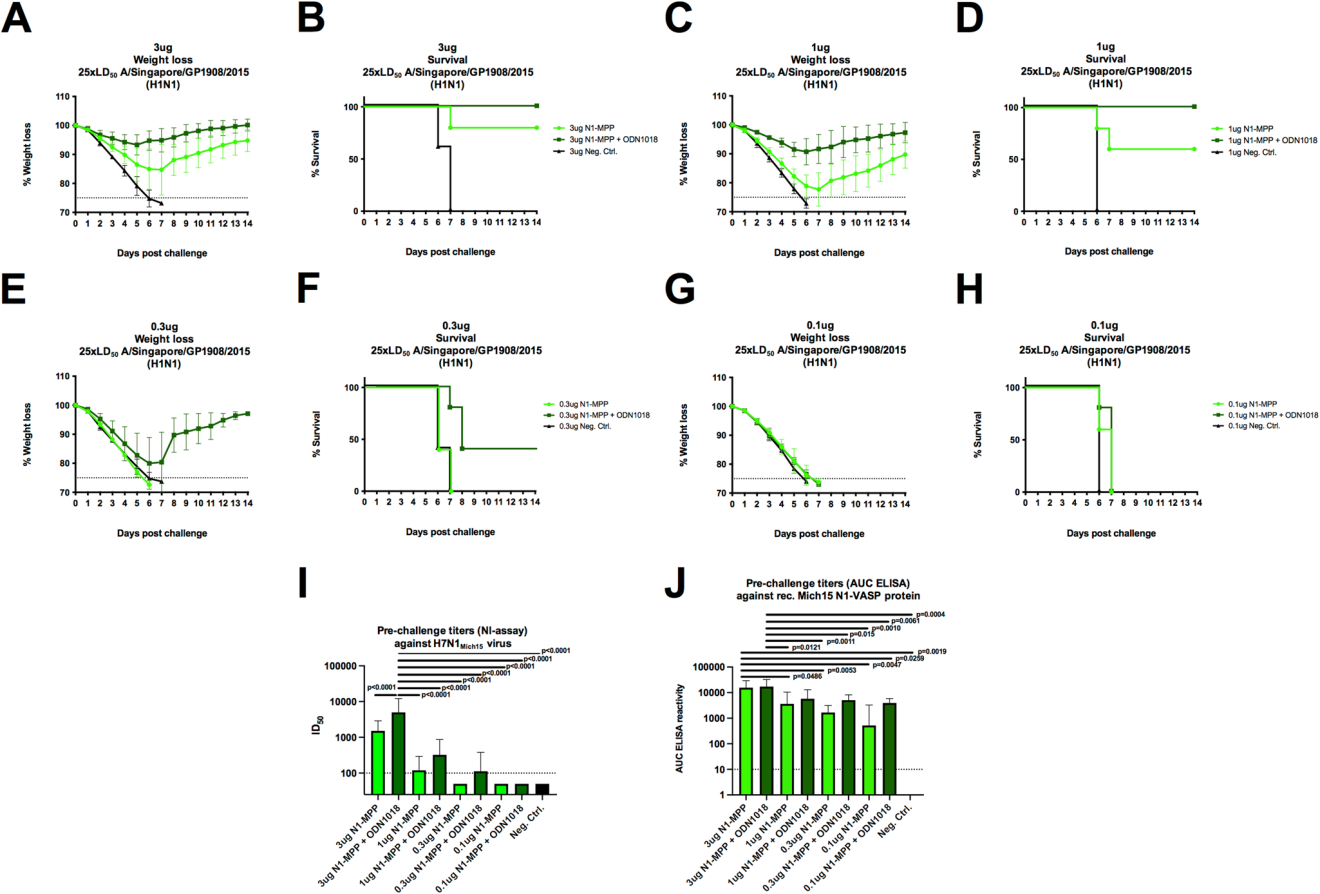
Dose testing of N1-MPP. Mice (*n* = 4–5 per group) were vaccinated in a prime/boost regimen with different doses of antigen (3 µg, 1 µg, 0.3 µg, 0.1 µg) and then challenged with 25xmLD_50_ of A/Singapore/GP1908/15 H1N1 (IVR-180). Weight loss (mean plus standard deviation) and survival were monitored over a 14-day period. Blood was obtained 42 days after prime and serological testing via ELISA and an NI assay was performed. **A**, **C**, **E**, **G** Weight loss and **B**, **D**, **F**, **H** survival curves for the respective dose groups. Differences in survival were analyzed using a Mantel–Cox log-rank test. 3 µg N1-MPP vs 3 µg Neg. Ctrl. *p* = 0.0116; 3 µg N1-MPP + ODN1018 vs 1 µg Neg. Ctrl. *p* = 0.0031; 1 µg N1-MPP vs 1 µg Neg. Ctrl. *p* = 0.0143; 1 µg N1-MPP + ODN1018 vs 1 µg Neg. Ctrl. *p* = 0.0027; 0.1 µg N1-MPP vs 0.1 µg Neg. Ctrl. *p* = 0.0495; 0.1 µg N1-MPP + ODN1018 vs 0.1 µg Neg. Ctrl. *p* = 0.0143; the differences between the remaining curves were not statistically significant (*p* > 0.05). **I** NI using H7N1_Mich15_ virus and **J** ELISA against rec. Mich15 N1-VASP protein with serum from mice vaccinated for the dose de-escalation experiment. Statistical analysis in **I** and **J** was performed using an one-way ANOVA corrected for multiple comparisons, shown is mean plus standard deviation.

In the previous animal experiments described above, a standard amount of 3 μg N1-MPP protein per vaccine dose was used in a prime-boost regimen. Using this quantity of NA protein, it was observed that mice vaccinated with unadjuvanted N1-MPP, experienced approximately 10% weight loss (Fig. 5A), with one mouse which succumbing to infection on day 7 post challenge (Fig. 5B). However, mice which received N1-MPP + CpG 1018 did not experience any significant morbidity or mortality. To assess if this trend could be maintained with a reduced amount of N1-MPP, we performed a dose de-escalation study. Mice (*n* = 4–5 per group) were vaccinated IM in a prime/boost regimen with different amounts of antigen and challenged three weeks after the boost with 25xmLD_50_ of A/Singapore/GP1908/15 H1N1 (IVR-180). Mice were vaccinated with 1 µg, 0.3 µg, or 0.1 µg of N1-MPP, N1-MPP + CpG 1018 or an irrelevant protein. Mice which received 1 μg of N1-MPP experienced around 20% weight loss (Fig. 5C), with two mice succumbing to infection on day 6 and 7 post challenge (Fig. 5D). However, the 1 μg N1-MPP + CpG 1018 group experienced only 5% weight loss and showed full protection. When vaccinated with 0.3 μg of non-adjuvanted N1-MPP, all mice succumbed to infection by day 7 similar to the negative control group (Fig. 5E, F). The 0.3 μg N1-MPP + CpG 1018 group experienced high weight loss of almost 20% and three mice succumbed to infection by day 8. Vaccination with 0.1 μg of antigen, no matter if adjuvanted or non-adjuvanted, failed to protect mice against viral challenge and all mice succumbed to infection by day 7 (Fig. 5G, H). In terms of serological responses, there was a high level of N1-specific antibodies detectable via ELISA (against Mich15 N1-VASP protein) in mice which received 3 μg (Fig. 5C) and 1 μg (Fig. 5G) of antigen. Even though vaccination with 0.3 μg (Fig. 5K) and 0.1 μg (Fig. 5J) failed to induce protection in vivo, serological analysis revealed that there was still an N1 antibody response detectable, albeit only at low levels. In terms of NI activity, there were bigger differences detected between individual dose groups than in ELISA. After vaccination with 3 μg of N1-MPP + CpG 1018, the ID_50_ was 5000 (geometric mean) (Fig. 5I). Once the dose was reduced to 1 µg, the ID_50_ dropped to 324 for the N1-MPP + CpG 1018 group and to 119 for the non-adjuvanted N1-MPP group (Fig. 5I). For mice that received 0.3 μg of antigen, the N1-MPP + CpG 1018 group showed an ID_50_ of 113 (Fig. 5I). In the case of the 0.1 μg group, the ID_50_ level decreased to below the limit of detection (Fig. 5I). In general, while binding antibodies were present, these antibodies had little to no NI activity in groups which received low amounts of NA protein.

### N2-MPP and B-NA-MPP form stable tetramers and exhibit full enzymatic activity

**Fig. 6:**
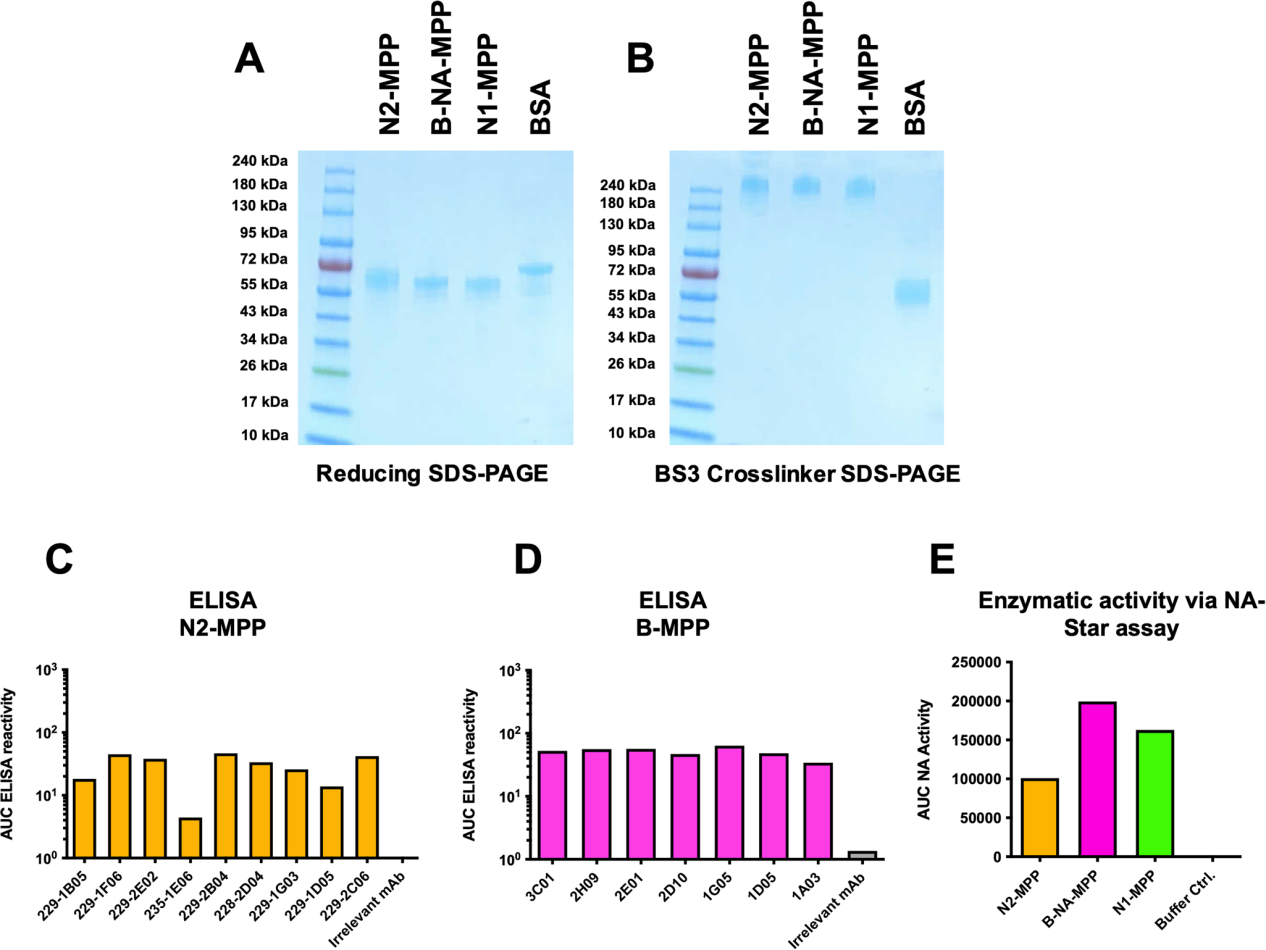
Structural analysis of N2-MPP and B-NA-MPP. **A** SDS-PAGE under denaturating conditions, all proteins show monomeric structures at an expected size of ~60 kDa. BSA was included as a monomer control. **B** SDS-PAGE using a BS3 crosslinker. N1-MPP, N2-MPP and B-NA-MPP show tetrameric structures at around 240 kDa. BSA was included as a monomer control. **C** ELISA against rec. N2-MPP using a broad panel of human anti-N2 mAbs to verify correct presentation of epitopes. **D** ELISA against rec. B-NA-MPP using a broad panel of human anti-B-NA mAbs to verify correct presentation of epitopes. **E** Enzymatic activity of N1-MPP, N2-MPP, B-NA-MPP was determined via NA-Star assay. Assays in **C** to **E** were run once in duplicates and the duplicates were used to calculate one area under the curve (AUC) value.

Current circulating influenza viruses in humans include H1N1, H3N2 and influenza B viruses. Therefore, we generated N2-MPP and B-NA-MPP constructs in addition to the N1-MPP construct. We cloned the sequences encoding for N2 of A/Kansas/14/17 (H3N2) and influenza B-NA of B/Colorado/6/17 into a pFastBac Dual vector containing a measles virus phosphoprotein tetramerization domain^10^. The constructs were expressed in insect cells and purified via an N-terminal hexahistidine tag. Structural integrity of the proteins was verified by visualizing them on a sodium dodecyl-sulfate polyacrylamide gel electrophoresis (SDS-PAGE). Under reducing conditions, N1, N2, and B-NA-MPP were visible as monomers at an expected size of ~60 kDa (Fig. 6A). By adding BS3 crosslinker, which cross-links primary amines, it was possible to confirm successful tetramerization of the N2-MPP and the B-NA-MPP protein. The tetramers were detectable at a size of ~240 kDa (Fig. 6B), which is comparable to the already established N1-MPP construct which was included as a control. Bovine serum albumin (BSA) served as monomeric control. To confirm that the NA-MPP proteins present the correct antigenic epitopes, an ELISA was performed using a broad panel of N2 (Fig. 6C)^15^ and B-NA (Fig. 6D)^16^ specific human monoclonal antibodies (mAbs). An irrelevant anti-Lassa antibody KL-AV-1A12 was included as negative control. All mAbs showed strong binding to N2-MPP and B-NA-MPP suggesting that the probed epitopes are presented in a native-like conformation. Next, we wanted to determine if the proteins are enzymatically active. For this, a standard NA-Star assay was performed. N1-MPP was included as a positive control. It was observed that N2-MPP and B-NA-MPP had high enzymatic activity comparable to the already established N1-MPP construct (Fig. 6E). However, the NA activity significantly varied between N1, N2, and B-NA-MPP which is not surprising since the different NA subtypes are known to have varying enzymatic activity based on the strain of origin^17^.

### Vaccination with recombinant N2-MPP and B-NA-MPP provides full protection against lethal influenza virus challenge in the mouse model

**Fig. 7:**
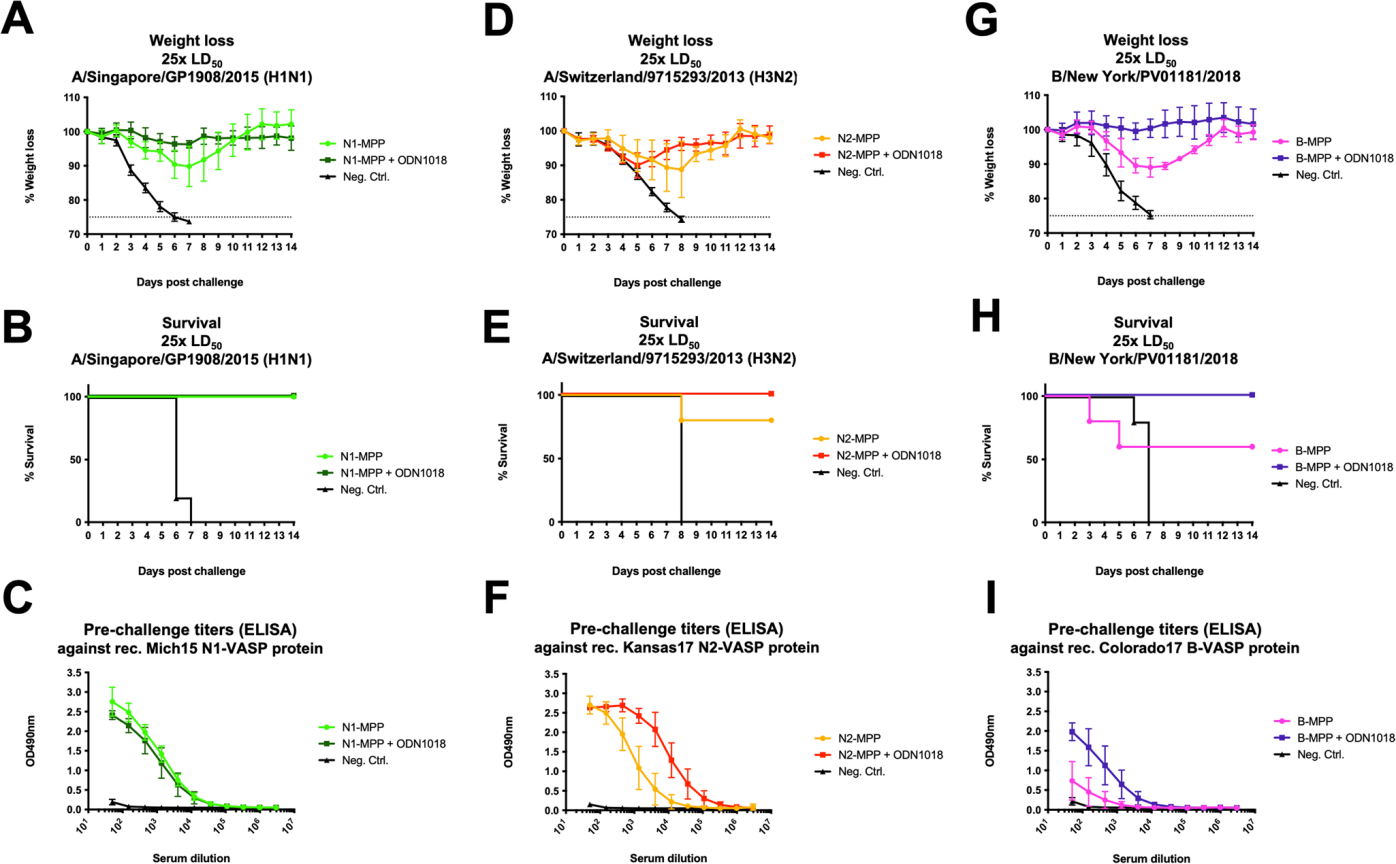
Testing the protective potential of N2-MPP and B-NA-MPP in vivo. Female 6–8 week old BALB/c (**A**–**C, G**–**I**) or DBA.2 (**D–F**) mice (*n* = 5 per group) were vaccinated in a prime/boost regimen with the respective recombinant protein. Blood was obtained 42 days after the prime and used for serological analysis. **A** Weight loss curve and **B** survival curve after challenge with 25xmLD_50_ of A/Singapore/GP1908/15 H1N1 (IVR-180). Differences in survival were analyzed using a Mantel–Cox log-rank test. N1-MPP vs Neg. Contr. *p* = 0.0035; N1-MPP + ODN1018 vs Neg. Contr. *p* = 0.0035; other differences were not statistically significant (*p* > 0.05). **C** ELISA against rec. Mich15 N1-VASP protein. **D** Weight loss curve and **E** survival curve after challenge with 25xmLD_50_ of A/Switzerland/9715293/13 (H3N2, mouse adapted). Differences in survival were analyzed using a Mantel–Cox log-rank test. N2-MPP vs Neg. Contr. *p* = 0.0143; N2-MPP + ODN1018 vs Neg. Contr. *p* = 0.0027; other differences were not statistically significant (*p* > 0.05). **F** ELISA against recombinant N2-VASP protein. **G** Weight loss and **H** survival after challenge with 25xmLD_50_ of B/New York/PV01181/18. Differences in survival were analyzed using a Mantel–Cox log-rank test. B-MPP + ODN1018 vs Neg. Contr. *p* = 0.0035; other differences were not statistically significant (*p* > 0.05). **I** ELISA was performed using serum samples against the rec. B-NA-VASP protein. A, C, D, F, G and I: shown is mean plus standard deviation.

To test if the recombinant N2-MPP and B-NA-MPP could induce a protective immune response, we vaccinated mice IM with 3 μg of the respective antigen (*n* = 5 per group) in a prime-boost regimen. The protein was either given non-adjuvanted or supplemented with CpG 1018. Irrelevant protein was administered as negative control. Vaccination with N1-MPP was included as a positive control. Following vaccination, mice were challenged either with 25xmLD_50_ of A/Singapore/GP1908/15 (H1N1), A/Switzerland/9715293/13 (H3N2, mouse adapted) or B/New York/PV01181/18. Mice vaccinated with N1-MPP alone experienced a weight loss of around 10%, whereas the N1-MPP + CpG 1018 group did not show any morbidity or mortality (Fig. 7A, B). In case of N2-MPP vaccination, both the non-adjuvanted and adjuvanted group showed ~10% weight loss (Fig. 7D). However, the N2-MPP + CpG 1018 group did not experience any mortality, whereas in the N2-MPP group one mouse succumbed to infection on day 8 post challenge (Fig. 7E). For the B-NA-MPP vaccination, the unadjuvanted group experienced 10% weight loss, with two mice succumbing to infection on day 3 and 5 post challenge (Fig. 7G, H). However, the B-NA-MPP + CpG 1018 group did not show any morbidity or mortality. The antibody response against the individual NA-MPP antigens was tested via ELISA. In general, non-adjuvanted groups induced a lower immune response compared to the groups receiving NA protein with CpG 1018 (Fig. 7C, F, I).

### A trivalent NA-MPP vaccine formulation does not induce antigenic competition between the individual NAs and is capable of inducing a strong immune response

**Fig. 8:**
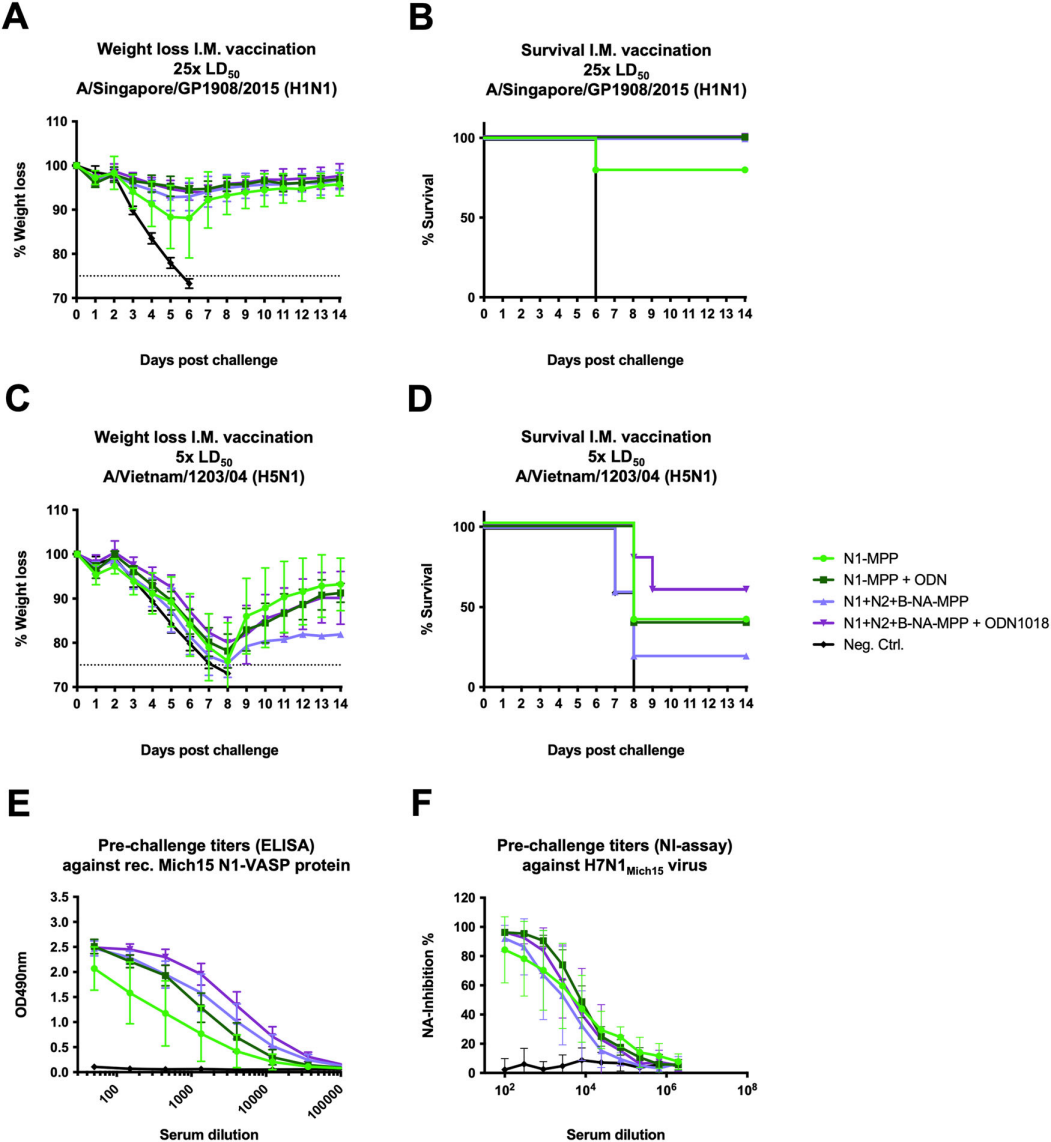
In vivo testing of a trivalent NA-MPP vaccine. Female, 6–8 week old BALB/c mice (*n* = 5 per group) were vaccinated in a prime/boost regimen with the respective antigens. Blood was obtained 42 days after the prime and used for serological analysis. **A** Weight loss and **B** survival curve after challenge with 25xmLD_50_ of A/Singapore/GP1908/15 H1N1 (IVR-180). Differences in survival were analyzed using a Mantel–Cox log-rank test. N1-MPP vs Neg. Ctrl. *p* = 0.0143; all other vaccinated groups vs Neg. Ctrl. *p* = 0.0027; other differences were not statistically significant (*p* > 0.05). **C** Weight loss and **D** survival curve after challenge with 5xmLD_50_ of A/Vietnam/1203/04 H5N1 (6:2 A/Puerto Rico/8/34 reassortant, polybasic cleavage site removed). Differences in survival were analyzed using a Mantel–Cox log-rank test. N1 + N2 + B-NA-MPP + ODN1018 vs. Neg. Ctrl. (*p* = 0.0116) was statistically significant, the differences between remaining curves were not statistically significant (*p* > 0.05). **D**, **E** ELISA against rec. Mich15 N1-VASP protein. **F** NI using the reassortant virus H7N1_Mich15_. **A**, **C**, **E,** and **F** are shown as mean plus standard deviation.

Since H1N1, H3N2 and influenza B viruses are all circulating in humans, a trivalent vaccine formulation containing all three NAs would be necessary for protection against all three types of viruses. For this reason, mice (*n* = 5 per group) were vaccinated either with N1-MPP, N1-MPP + CpG 1018, N1 + N2 + B-NA-MPP, N1 + N2 + B-NA-MPP + CpG 1018 or influenza B virus HA protein (negativ control) in a prime-boost regimen. After vaccination, mice were challenged with 25xmLD_50_ of A/Singapore/GP1908/15 (H1N1). The group which received N1-MPP alone experienced ~10% weight loss with one mouse which succumb to infection on day 6 (Fig. 8A, B). The other groups did not show any morbidity or mortality. To test if vaccination would also protect against challenge with a heterologous N1 virus, another subset of mice was challenged with 5xmLD_50_ of A/Vietnam/1203/04 (H5N1, 6:2 A/Puerto Rico/8/34 reassortant, HA polybasic cleavage site removed). While all groups experienced weight loss of ~20%, partial protection was observed in all vaccinated groups with the trivalent, adjuvanted formulation performing best (Fig. 8C, D). In terms of serology, mice which had been vaccinated with N1 + N2 + B-NA-MPP + CpG 1018 had the strongest antibody response in ELISA against recombinant Mich15-VASP protein (Fig. 8E). In an NI assay using H7N1_Mich15_ the inhibition potential of groups N1-MPP + CpG 1018 and N1 + N2 + B-NA-MPP + CpG 1018 was the highest (Fig. 8F).

## Discussion

Influenza virus vaccines provide significant protection against influenza virus infections but the current vaccines are impacted by antigenic drift^18–22^. In addition, seasonal influenza virus vaccines induce an unbalanced immune response mostly targeting the HA but not the NA of the virus^6,15^. However, NA undergoes somewhat slower antigenic drift^8^ and anti-NA immunity can be highly protective in animal models and humans^1–6,9,11,23–28^. Several factors may influence this lack of immunogenicity of NA in seasonal vaccines, including non-standardized amounts of NA in the vaccine preparations, instability of conformational epitopes on the NA in the formulations, and HA immunodominance over NA. Vaccination with stable, tetrameric recombinant NA could overcome these issues, either when used as a standalone vaccine or when admixed to current seasonal vaccines. We have previously reported recombinant NA constructs based on N1 NA fused to a measles virus phosphoprotein tetramerization domain^10^. Here we have expanded this work to N2 and influenza B virus NA and have shown that these proteins are also immunogenic and protective in the mouse model, especially when used with a TLR9 adjuvant, CpG 1018. Importantly, mixing these three components into a trivalent formulation did not decrease the immune response to the N1 component or protection from challenge with H1N1 or H5N1 viruses. Through passive transfer experiments we also demonstrated that the induced humoral immune response is sufficient for protection, even though cellular immune responses to NA may contribute to protection as well. For initial experiments we used a 3 μg dose of recombinant NA. The rationale behind this was that split vaccine with 1 μg of HA per dose is often used by us and others in the mouse model. The human dose is 15 µg, so 1/15th of the human dose is used. Recombinant HA-based vaccines for humans contain 45 μg of HA per subtype and since the vaccine tested here is a recombinant protein vaccine, we used 1/15th of 45 μg for the recombinant NA as well, resulting in a 3 μg dose. In a dose de-escalation study performed here the 3 μg dose worked indeed better than lower doses. However, it is unclear if increasing the dose further would lead to an improvement in terms of protection. For humans, we are planning to give only one dose (because they are primed) and we will not test doses above 45 μg rNA per shot since we do not think that higher amounts of recombinant NA per dose would be commercially feasible.

The main focus of this study was to explore the combination of an adjuvant with our recombinant NA constructs. The TLR9 agonist CpG 1018 was selected as adjuvant since it has an extensive safety record and is currently used in a licensed hepatitis B virus vaccine^13,14^ which are important pragmatic reasons to include it as adjuvant and which facilitate clinical development significantly. Here we show, that CpG 1018 has a significant adjuvant effect in terms of the induced immune response, protection from the challenge as well as in terms of antigen sparing. In addition, we detected strong crossreactivity to heterologous N1 NAs, especially in the adjuvanted groups, and the adjuvanted trivalent NA vaccine showed the highest degree of protection against H5N1 challenge. The adjuvanted recombinant NA vaccine consistently outperformed the non-adjuvanted recombinant NA vaccine in our study. Previously, we have shown that even when recombinant NA is admixed to seasonal vaccine preparations, HA dominates over NA and suppresses a robust anti-NA response^10^. By administering seasonal vaccine into one leg of the mice and recombinant NA into another leg this could be partially circumvented and led to a strong NA response. However, this is of course less practical for a vaccine product. Here we observed that the addition of CpG 1018 to the seasonal vaccine/recombinant NA admixture at least partially broke the immunodominance of the HA, making it possible to just administer one shot that still resulted in robust anti-NA immunity. The mechanism behind this is so far unclear but could be as simple as attracting more immune cells to the injection side and providing an innate immune trigger that enhances the immune response in general. If this effect is unique to CpG 1018 remains to be determined. In the future, we plan to explore this and also conduct an analysis of innate and T-cell immune responses after vaccination with adjuvanted recombinant NA immunogens. Of course, an important caveat of our study which needs to be pointed out is, that it was performed in naive mice and that so far we have not tested the vaccine candidate in other animal models like hamsters and ferrets. Humans are already immunologically primed for NA and will therefore likely respond differently than naive mice.

In summary, we show that the combination of recombinant NA with CpG 1018 is inducing robust anti-NA immunity and protection in the mouse model. This warrants further clinical development of the combination and will hopefully result in a next-generation seasonal influenza virus vaccine with more resistance to antigenic drift, which may also partially protect from emerging pandemic influenza virus subtypes.

## Methods

### Cells and viruses

Madin-Darby canine kidney (MDCK) cells (ATCC #CCL-34) were maintained in Dulbecco’s Modified Eagle’s medium (DMEM; Gibco) containing 10% fetal bovine serum (FBS; Gibco), 1% penicillin/streptomycin antibiotics mix (100 U/ml penicillin, 100 µg/ml streptomycin; Gibco) and 1% hydroxyethylpiperazine ethane sulfonic acid (HEPES; Gibco). BTN-TN-5B1-4 (*Trichoplusia ni*, High Five) cells were maintained in Express Five media (Gibco) containing 1% L-glutamine (Gibco) and 1% penicillin/streptomycin antibiotics mix. Sf9 (*Spodoptera frugiperda*) cells were maintained in *Trichoplusia ni* medium – Fred Hink (TNM-FH; Gemini Bioproducts) containing 10% FBS, 1% penicillin/streptomycin antibiotics mix, and 1% Pluronic F-68 (Sigma Aldrich). For passaging of baculovirus stocks in Sf9 cells, the medium was switched to TNM-FH containing 3% FBS, 1% Pluronic F-68 and 1% penicillin/streptomycin antibiotics mix. The reassortant viruses used in this study were grown in 10-day-old embryonated chicken eggs (Charles River Laboratories). The H7N1 viruses used in NA inhibition assay, contain the internal genes of A/Puerto Rico/8/34 H1N1 an exotic H7 HA of A/mallard/Alberta/24/01 H7N3 and either the N1 of A/Michigan/45/2015 H1N1 (H7N1_Mich15_) or A/California/04/09 H1N1 (H7N1_Cal09_). The challenge virus A/Singapore/GP1908/15 (H1N1, IVR-180 strain) possesses the internal proteins of A/Texas/1/77 (H3N2) and the surface glycoproteins of A/Singapore/GP1908/15 (pH1N1). A/Switzerland/9715293/13 (H3N2) and B/New York/PV01181/18 are based on wild type backbones but are mouse-adapted; A/Vietnam/1203/04 (H5N1) is a reassortant virus with the internal genes of A/Puerto Rico/8/34 H1N1 and has a deleted polybasic cleavage site.

### Recombinant proteins

Recombinant proteins used in this study were generated by using the baculovirus expression system. Briefly, coding sequences for N1-MPP, N2-MPP and B-MPP were cloned into a modified pFastBac vector. The vectors where then transformed into DH10Bac, appropriate clones were picked based on blue/white screening, the clones were grown and midiprepped and the resulting bacmids were transfected into Sf9 cells for baculovirus rescue. Rescued baculovirus was then propagated in Sf9 cells and used to infect High Five cells for protein expression at a multiplicity of infection of 10. Three days post infection, the High Five cell supernatant was harvested and recombinant NAs were purified using Ni^2+^ chelate chromatography. The recombinant N1-MPP, N2-MPP and B-MPP proteins used for animal vaccination studies are structured into an N-terminal signal peptide, followed by a hexahistidine purification tag, a measles virus phosphoprotein tetramerization domain, a thrombin cleavage site and either the N1 globular head domain (A/Michigan/45/15), the N2 globular head domain (A/Kansas/14/17 (H3N2)) or an influenza B NA globular head domain (B/Colorado/6/17)). Influenza B virus HA of B/Malaysia/2506/04 (B-Mal-HA) served as a negative control except for experiments with an influenza B virus challenge where a Lassa glycoprotein was used instead, the constructs were designed as described previously. The recombinant NA proteins of A/New Caledonia/20/99 H1N1 (NC99), A/Puerto Rico/8/34 H1N1 (PR8), A/Brisbane/02/18 H1N1 (Bris18), A/California/04/09 (Cal09) H1N1, A/Vietnam/1203/04 H5N1 (Vn04), A/Kansas/14/17 H3N2 (Kansas17), B/Colorado/6/17 (Colorado17) and A/Michigan/45/15 H1N1 are designed in the same way as the N1-MPP construct with the difference that they contain a vasodilator stimulated phosphoprotein (Mich15-VASP) tetramerization domain instead of a MPP domain. Protein concentrations were measured using Quick Start™ Bradford 1× Dye Reagent (BioRad). The proteins were stored at −80 °C.

### Sodium dodecyl-sulfate polyacrylamide gel electrophoresis (SDS-PAGE)

To confirm protein integrity, an SDS-PAGE was performed under reducing conditions and by using a bis-sulfosuccinimidyl suberate (BS3; ThermoFisher) crosslinker. For the BS3 crosslinker SDS-PAGE, the proteins were treated with the crosslinker according to the manufacturer’s instruction. For the SDS-PAGE 1.5 μg of the respective NA protein was mixed 1:1 with 2× Laemmli loading buffer (BioRad) supplemented with 5% beta-mercaptoethanol. The samples were then heated for 10 minutes at 95 °C prior to loading them on a sodium dodecyl-sulfate polyacrylamide gel (4–20% Mini-PROTEAN^®^TGX™ Precast Protein Gels, BioRad). Afterwards, the gels were stained with Coomassie blue (ThermoFisher) for 1 hour at room temperature and destained with distilled water to visualize the proteins. BSA was used as a monomeric control.

### Animal work

All animal experiments were performed under protocols approved by the Icahn School of Medicine at Mount Sinai Institutional Animal Care and Use Committee. For all animal experiments conducted, female 6–8 week old BALB/c or DBA.2 mice (Jackson laboratories, *n* = 5 per group) were used, unless otherwise mentioned. The adjuvant, CpG 1018 (TLR9 agonist), used in this study was provided by Dynavax Technologies and administered at a dose of 3 μg per mouse. Seasonal QIV used in these experiments was Flucelvax (LOT 252380, season 2017/18). To observe if two vaccinations are required to induce a robust protective immune response, mice were primed intramuscularly (IM) or primed and boosted (with a 3-week interval) with 3 μg N1-MPP, 3 µg N1-MPP + 3 μg CpG 1018 or 3 µg of an irrelevant protein (B-Mal-HA). Six weeks after the prime, mice were intranasally challenged with 10× the 50% mouse lethal dose (mLD_50_) of A/Singapore/GP1908/15 H1N1 (IVR-180). Weight loss and survival were monitored over a period of 14 days. Mice were euthanized if they lost more than 25% of their initial body weight. Blood was taken from each mouse on day 21 and day 42 post prime.

In addition, a passive serum transfer was performed using serum from mice previously vaccinated with N1-MPP, N1-MPP + CpG 1018 and B-Mal-HA. Naive mice received 200 μl of serum intraperitoneally. After 2 hours, the mice were intranasally challenged with 5xmLD_50_ of A/Singapore/GP1908/15 H1N1 (IVR-180) and weight loss and survival were monitored over 14 days.

To assess the effect of CpG 1018 in more detail, mice were vaccinated IM in a follow up experiment in a prime/boost regimen separated into the following groups: 3 µg N1-MPP, 3 µg N1-MPP + 3 μg CpG 1018, QIV (1 µg of each HA), QIV + 3 μg CpG 1018, 3 µg N1-MPP admixed with QIV, 3 µg N1-MPP admixed with QIV and 3 μg CpG 1018, 3 µg N1-MPP given in the right leg and QIV given in the left leg at the same time (N1-MPP (r)/QIV (l)), N1-MPP + 3 μg CpG 1018 (r)/QIV + 3 μg CpG 1018 (l) or 3 µg B-Mal-HA. Mice were then challenged intranasally with 25xmLD_50_ of A/Singapore/GP1908/15 H1N1 (IVR-180). Weight loss and survival were monitored over 14 days. Blood was obtained on day 21 and day 42 after prime. Mice were euthanized if they lost 25% or more of their initial body weight.

To determine the viral load in murine lung tissues, mice were vaccinated using the same regimen but with the difference that they were challenged with a reduced challenge dose of 1xmLD_50_ of A/Singapore/GP1908/15 H1N1 (IVR-180) to gain a better resolution. Lungs were taken on day 3 and day 6 post challenge, homogenized and the viral titer determined via standard plaque assay^5^.

To assess if the amount of N1-MPP protein could be potentially reduced yet still induce a protective immune response, mice were vaccinated IM in a prime-boost regimen with different doses of N1-MPP. Mice received either 3 µg, 1 µg, 0.3 µg, or 0.1 µg of N1-MPP, N1-MPP + the matching amount of CpG 1018 or irrelevant B-Mal-HA protein. Mice were then challenged with 25xmLD_50_ of A/Singapore/GP1908/15 H1N1 (IVR-180) and weight loss and survival were monitored over 14 days. Mice were euthanized if they lost more than 25% of their initial body weight.

To observe the protective potential of N2-MPP and B-NA-MPP, mice were vaccinated IM in a prime-boost regimen with 3 μg N2/B-NA-MPP, 3 μg N2/B-NA-MPP + CpG 1018, or 3 μg B-Mal-HA (or Lassa glycoprotein for mice challenged with influenza B virus). Vaccination with N1-MPP in the same setup was included as a positive control. Mice were then either challenged with 25xmLD_50_ of A/Singapore/GP1908/15 H1N1 (IVR-180) or 25xmLD_50_ of B/New York/PV01181/18. For the challenge with 25xmLD_50_ of A/Switzerland/9715293/13 (H3N2) female DBA/2 J mice were used. Weight loss and survival were monitored over 14 days. Mice were euthanized if they lost more than 25% of their initial body weight. Blood was obtained on day 21 and day 42 after prime.

To determine if there would be antigenic competition between the individual NA-MPP proteins when combined in one vaccination, mice were vaccinated IM in a prime-boost regimen either with 3 μg N1-MPP, 3 μg N1-MPP + CpG 1018, 3 μg of each N1, N2 and B-NA-MPP, 3 μg of each N1, N2 and B-NA-MPP + CpG 1018 or 3 μg of B-Mal-HA. Mice were then challenged either with 25xmLD_50_ of A/Singapore/GP1908/15 H1N1 (IVR-180) or 5xmLD_50_ of A/Vietnam/1203/04 (H5N1, 6:2 A/Puerto Rico/8/34 reassortant, HA with deleted polybasic cleavage site). Weight loss and survival were monitored over 14 days. Mice were euthanized if they lost more than 25% of their initial body weight. Blood was obtained on day 21 and day 42 after prime.

### NA-star assay

The NA enzymatic activity of the NA-MPP proteins was determined by using the NA-Star™ Influenza NA Inhibitor Resistance Detection Kit (ThermoFisher) following the manufacturer’s instructions. As a starting concentration, 10ug/ml of the respective protein was used and then serially diluted 1:3 across the plate. The signal was based on luminescence read-out and was measured using a Synergy H1 hybrid multimode microplate reader (BioTek). The data were analyzed using GraphPad Prism 8.

### Enzyme-linked immunosorbent assay (ELISA)

ELISAs were conducted as described previously^12^. Briefly, flat-bottom 96-well plates (Immulon 4 HBX plates, ThermoFisher) were coated overnight at 4 °C with 50 µl/well of the respective recombinant protein at a concentration of 2 µg/ml diluted in phosphate-buffered saline (PBS, pH = 7.4; Gibco). On the next day, the coating solution was removed and the plates were blocked for 1 h at room temperature (RT) with 100 µl/well of 3% milk mixed with 0.1% Tween 20 (PBST). The blocking solution was discarded and the serum samples were diluted to a starting concentration of 1:50 in 1% milk/PBST followed by a 1:3 dilution across the plate. The samples were then incubated on the plate for 2 h at RT. For ELISAs which were performed using mAbs, the antibodies were diluted to a start concentration of 30 µg/ml and then serially diluted 1:3 across the plate. The antibodies were incubated on the plate for 1 h at RT. For the N2-MPP epitope testing a broad panel of human derived N2-specific mAbs was used including 229-1B05, 229-1F06, 229-2E02, 235-1E06, 228-2D04, 229-1G03, 220-1D05, 229-2C06^15^. In the case of the B-MPP epitope testing the human B-NA specific antibodies 3C01, 2H09, 2E01, 2D10, 1G05, 1D05, and 1A03^16^ were used. Afterwards, plates were washed three times with PBST and incubated for 1 h at RT with the secondary antibody anti-mouse IgG H&L peroxidase-conjugated (Rockland, 610-1302, used 1:3000) or anti-human IgG Fab-specific horseradish peroxidase (HRP) (Sigma Aldrich, A0293-1ML, used 1:5000). The antibody was diluted 1:3000 in 1%milk/PBST and 100 µl/well were added to the plate. The plates were washed again three times with PBST and then developed by adding 100 µl/well of SigmaFast o-phenylenediamine dihydrochloride (OPD) solution (Sigma Aldrich) for 10 minutes. The reaction was stopped by adding 50 µl/well of 3 M hydrochloric acid (HCl). The signal was read using a Synergy H1 hybrid multimode microplate reader (BioTek) at an optical density of 490 nm. The data were analyzed using GraphPad Prism 8 software and values were expressed as the area under the curve (AUC). The cutoff value was defined as the average of all blank wells plus three times the standard deviation of the blank wells.

### Neuraminidase inhibition (NI) assay

The NI assay was conducted as described previously^12^. Briefly, flat-bottom 96-well plates (Immulon 4 HBX plates, ThermoFisher) were coated overnight at 4 °C with 150 µl/well of fetuin (50 µg/ml; Sigma Aldrich). The next day, serum samples were heat inactivated for 1 h at 56 °C and then diluted to a starting concentration of 1:100 in PBS. The samples were diluted 1:2 across a fresh 96-well plate. The reassortant viruses used in this assay, H7N1_Cal09_ and H7N1_Mich15_, were diluted in PBS and then added to the serum dilution at 2× the 50% effective concentration (EC_50_) for 1 h 45 min shaking at RT. In the meantime, the fetuin coated plates were washed three times with PBST and blocked with 200 µl/well of 5% BSA/PBS for 1 h at RT. The plates were washed three times with PBST and 100 µl of the serum-virus mixture was transferred and incubated for 2 h at 37 °C. The plates were washed three times with PBST and 100 µl/well of peanut agglutinin conjugated to horseradish peroxidase (HRP) (PNA 5 µg/ml; Sigma Aldrich) were added for 1 h 45 at RT. The plates were washed three times with PBST and developed by adding 100 µl/well of SigmaFast OPD developing solution. The reaction was stopped after 7 minutes by adding 50 µl/well of 3 M HCl. The signal was measured by using a Synergy 4 plate reader at a wavelength of 490 nm and the individual inhibitory concertation 50 (IC_50_) was calculated using GraphPad Prism 8.

### Lung titers

Plaque assays for virus titration have been conducted as described previously^5^. Briefly, confluent MDCK monolayers were infected with different sample dilutions (1:10–1:1.000.000) of homogenized lung tissues diluted in 1× minimum essential medium (MEM) ((1% penicillin/streptomycin antibiotics mix, 1% HEPES, 1% L-glutamine and 1% sodium-bicarbonate (Gibco)) for 1 h at 37 °C. Afterwards, the virus dilution was removed and an overlay consisting out of 2% Oxoid agar (ThermoFisher), H_2_O, 2× MEM, diethylaminoethyl (0.1% (wt/vol) DEAE) dextran and N-p-Tosyl-L-phenylalanine chloromethyl ketone-treated trypsin (1 µg/ml) was added to the cells. Plates were incubated for 2 days at 37 °C and then fixed using 10% paraformaldehyde overnight at 4 °C. Afterwards, the agar overlay was carefully removed, and the plaques visualized by immunostaining. Plates were blocked for 1 h at RT with 3% milk/PBS. The blocking solution was discarded, and the plates incubated with primary antibody (anti-N1 mAb 4A5) diluted 1:3000 in 1% milk/PBS for 1 h at RT. The plates were washed three times with PBS and the secondary antibody (anti-mouse IgG H&L peroxidase-conjugated (Rockland)) was added for 1 h at RT. The plates were washed three times with PBS and then developed by adding KPL TrueBlue Peroxidase Substrate (SeraCare). The number of plaques was counted (*n* = 3 per group) and the virus titers presented as the log_10_ plaque forming units (PFU)/ml. The limit of detection for the assay was at 125 PFU/ml. The graphs were generated using GraphPad Prism 8.

### Statistical analysis

Titers were compared using a one-way analysis of variance corrected for multiple comparisons. Survival was compared using a Mantel–Cox log-rank test. All statistical analysis was performed in GraphPad Prism 9.0.1.

### Reporting summary

Further information on research design is available in the Nature Research Reporting Summary linked to this article.

## Supplementary information

REPORTING SUMMARY

## Data Availability

Underlying data are available from the corresponding author upon reasonable request.
