# Supplementary material for: A CpG 1018 adjuvanted neuraminidase vaccine provides robust protection from influenza virus challenge in mice
Source: NPJ Vaccines. 2022 Jul 22;7:81. doi: 10.1038/s41541-022-00486-w (PMC9305062; doi:10.1038/s41541-022-00486-w)
Supplement: Supplementary file 1 — REPORTING SUMMARY [file 41541_2022_486_MOESM1_ESM.pdf]

nature portfolio

Corresponding author(s): Florian Krammer

Last updated by author(s): 4/16/22

Reporting Summary

Nature Portfolio wishes to improve the reproducibility of the work that we publish. This form provides structure for consistency and transparency in reporting. For further information on Nature Portfolio policies, see our Editorial Policies and the Editorial Policy Checklist.

Statistics

For all statistical analyses, confirm that the following items are present in the figure legend, table legend, main text, or Methods section.

n/a	Confirmed	
☐	☒	The exact sample size (n) for each experimental group/condition, given as a discrete number and unit of measurement
☐	☒	A statement on whether measurements were taken from distinct samples or whether the same sample was measured repeatedly
☐	☒	The statistical test(s) used AND whether they are one- or two-sided<br>*Only common tests should be described solely by name; describe more complex techniques in the Methods section.*
☒	☐	A description of all covariates tested
☒	☐	A description of any assumptions or corrections, such as tests of normality and adjustment for multiple comparisons
☐	☒	A full description of the statistical parameters including central tendency (e.g. means) or other basic estimates (e.g. regression coefficient) AND variation (e.g. standard deviation) or associated estimates of uncertainty (e.g. confidence intervals)
☐	☒	For null hypothesis testing, the test statistic (e.g. F, t, r) with confidence intervals, effect sizes, degrees of freedom and P value noted<br>*Give P values as exact values whenever suitable.*
☒	☐	For Bayesian analysis, information on the choice of priors and Markov chain Monte Carlo settings
☒	☐	For hierarchical and complex designs, identification of the appropriate level for tests and full reporting of outcomes
☒	☐	Estimates of effect sizes (e.g. Cohen's d, Pearson's r), indicating how they were calculated

Our web collection on statistics for biologists contains articles on many of the points above.

Software and code

Policy information about availability of computer code

Data collection	No software was used.
Data analysis	GraphPad Prism 8 was used.

For manuscripts utilizing custom algorithms or software that are central to the research but not yet described in published literature, software must be made available to editors and reviewers. We strongly encourage code deposition in a community repository (e.g. GitHub). See the Nature Portfolio guidelines for submitting code & software for further information.

Data

Policy information about availability of data

All manuscripts must include a data availability statement. This statement should provide the following information, where applicable:

- Accession codes, unique identifiers, or web links for publicly available datasets
- A description of any restrictions on data availability
- For clinical datasets or third party data, please ensure that the statement adheres to our policy

Underlying data is available from the corresponding author upon reasonable request.

Field-specific reporting

Please select the one below that is the best fit for your research. If you are not sure, read the appropriate sections before making your selection.

☒ Life sciences ☐ Behavioural & social sciences ☐ Ecological, evolutionary & environmental sciences

For a reference copy of the document with all sections, see nature.com/documents/nr-reporting-summary-flat.pdf

Life sciences study design

All studies must disclose on these points even when the disclosure is negative.

Sample size	Sample sizes (n for animal numbers) were based on historical experience and numbers of animals that can be housed per cage (n=5 per group).
Data exclusions	Not data was excluded.
Replication	Experiments were performed once. However, experiments were replicated with small variations throughout the study (e.g. initial prime-boost mouse experiments versus admixture study versus dose escalation study versus monovalent/trivalent study) and yielded the same results.
Randomization	Naive animals were randomly assigned to cages. Cages were randomly assigned to groups.
Blinding	Blinding was not performed since most animal experiments were done by the same individuals (students).

Reporting for specific materials, systems and methods

We require information from authors about some types of materials, experimental systems and methods used in many studies. Here, indicate whether each material, system or method listed is relevant to your study. If you are not sure if a list item applies to your research, read the appropriate section before selecting a response.

Materials & experimental systems

n/a	Involved in the study	
☐	☒	Antibodies
☐	☒	Eukaryotic cell lines
☒	☐	Palaeontology and archaeology
☐	☒	Animals and other organisms
☒	☐	Human research participants
☒	☐	Clinical data
☒	☐	Dual use research of concern

Methods

n/a	Involved in the study	
☒	☐	ChIP-seq
☒	☐	Flow cytometry
☒	☐	MRI-based neuroimaging

Antibodies

Antibodies used	The following mAbs were used. For N2-MPP testing: 229-1B05, 229-1F06, 229-2E02, 235-1E06, 228-2D04, 229-1G03, 220-1D05, 229-2C06. In case of the B-MPP epitope testing the human B-NA specific antibodies 3C01, 2H09, 2E01, 2D10, 1G05, 1D05 and 1A03 were used.
Validation	Detailed mAb validation is described in:<br><br>Chen, Y. Q. et al. Influenza Infection in Humans Induces Broadly Cross-Reactive and Protective Neuraminidase-Reactive Antibodies. Cell 173, 417-429.e410, doi:10.1016/j.cell.2018.03.030 (2018).<br><br>Madsen, A. et al. Human Antibodies Targeting Influenza B Virus Neuraminidase Active Site Are Broadly Protective. Immunity 53, 852-863.e857, doi:10.1016/j.immuni.2020.08.015 (2020).

Eukaryotic cell lines

Policy information about cell lines

Cell line source(s)	Madin-Darby canine kidney (MDCK) cells were sourced from (ATCC #CCL-34).
Authentication	None of the cells used were authenticated.
Mycoplasma contamination	The cell line used tested negative for mycroplasma.

Commonly misidentified lines (See ICLAC register)	None.

Animals and other organisms

Policy information about studies involving animals; ARRIVE guidelines recommended for reporting animal research

Laboratory animals	6-8 week old female BALB/c and DBA.2 mice were used for all experiments.
Wild animals	The study did not involve wild animals.
Field-collected samples	The study did not involve field-collected samples.
Ethics oversight	All animal experiments were performed under protocols approved by the Icahn School of Medicine at Mount Sinai Institutional Animal Care and Use Committee.

Note that full information on the approval of the study protocol must also be provided in the manuscript.
